# The multi-faceted usage patterns of nutrition apps: a survey on the appropriation of nutrition apps among German-speaking users of MyFitnessPal

**DOI:** 10.1186/s12911-020-01294-9

**Published:** 2020-10-28

**Authors:** Paula Stehr, Veronika Karnowski, Constanze Rossmann

**Affiliations:** 1 Department of Media and Communication Science, University of Erfurt, Erfurt, Germany; 2 Department of Media and Communication, LMU Munich, Munich, Germany

**Keywords:** Nutrition apps, Mobile apps, mHealth, Appropriation, MPA model

## Abstract

**Background:**

Current systematic reviews of randomized controlled trials suggest positive influences of mobile app-based health promotion programs on dietary and physical activity behaviors. However, the actual adoption of and rates of nutrition app use remain low among the overall population. Therefore, we took a step back and investigated actual use patterns. In doing so, we took an appropriation perspective in order to reveal different styles of everyday life integration of these apps.

**Methods:**

We conducted an online survey with 761 German-speaking users of the MyFitnessPal app. Respondents were asked about their detailed use of nutrition apps according to an adapted version of the mobile phone appropriation model. Based on a cluster analysis, different user types were identified. These user types were compared and further described based on analyses of variance. In addition, we conducted a multinomial logistic regression to determine significant predictors of the varying usage patterns.

**Results:**

Four different user types were identified: (1) Supported, (2) Indifferent, (3) Health-conscious, and (4) Socializer. These user types mainly differed regarding three aspects: (1) their willingness to adjust default settings to one’s own needs and abilities, (2) the role of social support and social norms, and (3) app use for socializing and competition.

**Conclusions:**

Our study sheds light on the multi-faceted appropriation patterns of nutrition apps in Germany, thus paving the way for future studies on mHealth appropriation patterns and the design of more refined mHealth-interventions.

## Background

Due to huge coverage, mass media campaigns are mainly capable of imparting knowledge concerning health-related issues, but their effects regarding actual behavioral changes remain limited [1]. At the same time, the number of smartphone users is continuously increasing with the global mobile internet penetration having reached 47% in 2018 [2]. More and more often, scholars therefore suggest mobile media as a means to close this gap, in particular through the use of mobile apps [3, 4]. This suggestion originates with the deep embedding of mobile phones and in particular smartphones into the everyday lives of their users, constantly accompanying them throughout their daily lives [5] and thus, enabling them to continuously collect personal health data [6]. The potential of mobile apps to influence health-related behavioral changes is further enhanced by the fact that internet-enabled mobile devices do add a second layer to users’ everyday lives; being permanently online, users are constantly connected to and embedded in their social networks [5, 7–9], making it possible to share personal (health-related) progress and setbacks with peers and others, such as physicians [6].

A current systematic review suggests positive influences of mobile app-based health promotion programs on health outcomes, including diet and physical activity [10]. However, merely assessing effects in randomized controlled trials, effect studies typically lack external validity with regards to actual (non-forced) use behavior. Indeed, the adoption and continued use rates among the overall population remain low, especially with regards to nutrition apps [11]. We therefore took a step back and investigated the actual usage patterns of health apps. In doing so, we took an appropriation perspective in order to reveal different styles of everyday life integration beyond the mere adoption of these apps. That is, we investigated not only whether people use health apps or not and what the characteristics of users are, but we wanted to shed light on how they actually use health apps. As long as we do not understand how and why individuals use nutrition apps, which motives, perceptions, and expectations drive their usage, and how stable their usage patterns are, our knowledge concerning the role of nutrition apps in health promotion will remain limited.

We focus on nutrition apps as nutrition is a highly relevant issue with 39% of adults worldwide being overweight [12], and overweight and obesity costing healthcare systems worldwide an estimated US$500 billion per year [13]. Hence, based on an online survey of 761 users of one of the most popular nutrition apps (MyFitnessPal), we investigated patterns of continued nutrition app usage among German-speaking users.

### Use and effectiveness of nutrition apps

Research on nutrition apps so far has mainly focused on assessing their effectiveness, leading to mixed results [e.g., see references 14, 15]. A recent systematic review including studies on the effectiveness of nutrition apps revealed that only seven of the 13 studies identified reported positive outcomes [16]. In addition, many of these studies only investigated apps designed specifically for these studies [17], hence only investigating “whether their particular style of a black box application works better than not having any black box application” [18, p. 2].

Given this unsatisfactory state of research on nutrition app effectiveness, it seems reasonable to first take a step back and consider what we actually know about nutrition app usage. So far, only very few studies have focused on the actual specifics of usage of nutrition apps with many studies rather focusing on health app use in general as pointed out by König et al. [11]. These studies mostly analyze health app adoption or compare users with non-users, often providing contradictory evidence on differences in age, education, or health status [see 19–23]. One notable exception is the study by König et al. [11] in which a stage model approach to explain adoption of nutrition apps is used. The authors identified differences among users in these different stages and derive suggestions for targeted interventions based on these different stages.

However, despite this more nuanced view, the authors also focus on the mere adoption of nutrition apps. Adoption, however, is only one facet of use. Referring to Rogers’ [24] innovation-decision process adoption is only one stage in this process that deals with the binary question of use versus nonuse (i.e., adoption vs. rejection). Yet, after this decision, the implementation stage of an innovation (e.g., a nutrition app) follows, which arguably has even greater impact on its potential effectiveness. In this stage of implementation, questions of everyday-life integration are negotiated, and users’ specific use patterns are molded.

### An appropriation perspective: the MPA model

**Fig. 1:**
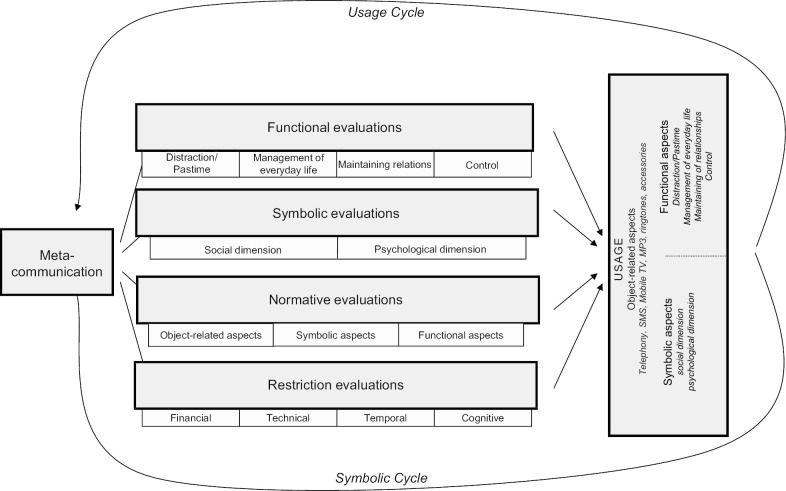
The MPA model (Wirth et al. [25], p. 606)

Hence, in order to go beyond this focus on the adoption of nutrition apps and to study actual prolonged use patterns as the basis of potential beneficial effects of nutrition app usage, we draw on the mobile phone appropriation model (MPA). This model was specifically developed to investigate the everyday life integration of mobile services [25]. In its basic structure, the MPA can be understood as an extension of the Theory of Planned Behavior (TPB) [26], explaining human behaviors through behavioral, normative, and control beliefs. However, in order to grasp additional aspects of mobile service appropriation, the model enhances this basic structure based on diffusion of innovations theory [24], the technology acceptance model [27], frame analysis [28], the domestication approach [29], and the uses-and-gratifications approach (UGA) [30]. The following section briefly explains MPA’s main concepts and assumptions (see Fig. 1).

Mainly drawing on the domestication approach [29], the MPA model considers appropriation to be a creative and active process that results in various usage patterns by individual users. This consideration is reflected in several behavioral sub-constructs in the model, for example, object-related and functional aspects. The first aspect comprises behaviors related to the device itself, such as the decoration of the device with accessories or the choice of a ringtone. The latter is informed by the UGA in which functional aspects, such as distraction/pastime or maintaining relations are differentiated. In addition to these aspects, the MPA model introduces symbolic aspects to the behavioral sub-constructs and takes into account the symbolic value of both the object and its usage. This symbolic aspect combines a social and a psychological dimension, for example, the aspects of behaviors important to the users themselves and the users in relation to their social surroundings. According to the classification of behaviors into the above-described subdimensions, behavioral beliefs are also differentiated into functional and symbolic evaluations along with the according subdimensions.

In addition, normative evaluations and restrictions are modeled to influence behavioral outcomes. Normative evaluations are split into the three behavioral aspects: (1) object-related, (2) symbolic, and (3) functional. Restriction evaluations are differentiated into four aspects: (1) financial, (2) technical, (3) temporal, and (4) cognitive. Furthermore, the MPA model introduces the concept of metacommunication into the appropriation process, i.e. the impact of communication about communication technologies. Three distinct forms of metacommunication are differentiated: (1) mass, (2) interpersonal, and (3) observation of others. In total the MPA model is conceptualized as a circular process with metacommunication discussing and influencing behavioral, normative, and control beliefs, which in turn influence behavioral aspects. These behavioral outcomes finally inform future metacommunication, thus constantly renewing the appropriation process.

So far, the MPA model has not only been applied to study the appropriation process of the innovation bundle mobile phone in total but in its basic principles and accordingly adapted forms also to study further areas and mobile applications, such as the mobile web [31], mobile Facebook use [32], or the relationship between mobile phone appropriation and migrant acculturation [33]. In the health domain, the model has already been applied to the study of diabetes apps and has unveiled their specific appropriation patterns [34]. Hence, as suggested by Wirth et al. [25], our first research question is:

#### RQ1

Based on the symbolic and functional aspects of the usage dimensions in the MPA model, which patterns of nutrition app appropriation can be found among German-speaking users?

In order to describe these patterns in more detail and mirroring the approach of König et al. [11] to describe different user groups, we also ask:

#### RQ2

How do these patterns differ in terms of app functionalities used and users’ sociodemographics?

Finally, in order to also accommodate the factors influencing actual usage behavior as modeled by the MPA model, we also ask:

#### RQ3

To what extent can these appropriation patterns be explained by metacommunication, functional, normative, and restriction evaluations?

## Methods

In order to answer these questions, we conducted an online survey in February and March 2015. The study focuses on the users of MyFitnessPal, a free application to track calorie intake and physical exercise. MyFitnessPal is one of the most popular lifestyle apps and the most common one in the field of nutrition [35, 36]. Beyond monitoring net calories, it also tracks major nutrients [37]. The link to the online survey was posted on the official German Facebook page “@myfitnesspalde” and blog of MyFitnessPal “blog.myfitnesspal.de”. Our final sample consisted of 761 respondents with 59.3% being female and 28.9% male. Some (11.8%) of the respondents did not reveal their gender. The respondents’ average age was 36.4 years (*SD* = 12.10). Furthermore, the sample consisted mainly of highly educated users (66.8%) who possessed a university entrance diploma or a university degree. Accordingly, 78.1% of respondents are currently employed. Also, with around two thirds having a net income of at least 1500 € per month, the income of our respondents was rather high at the time of data collection.

### Measurement

Our goal was to identify patterns of general nutrition app appropriation. Therefore, we asked participants to bear in mind not only MyFitnessPal but all different kinds of nutrition apps they may use. A key component of the questionnaire is an adapted version of the MPA scale [38], which is a scale specifically designed to measure the MPA model’s components. The MPA scale has already been validated in various studies [38, 39]. As suggested by von Pape et al. [38], questionnaire items were rephrased for the purpose of this study, and the subdimensions of functional aspects of usage adjusted in order to fit the object of investigation, the nutrition apps (see [40]).

According to Brown et al. [41], apps fulfill informational needs with their constant availability as a crucial aspect [42]. Hence, the possibility to continuously monitor and track information about one’s own health behaviors is considered one core aspect of health app usage [43]. We termed this first subdimension of functional aspects of usage *lifestyle management*. The items measuring this subdimension focus on the constant availability of general nutrition information and users’ nutrition status in addition to the general importance of maintaining a healthy lifestyle. Second, sharing of emotional states among like-minded people fosters attempts to improve health behavior in which some users consider competitive elements to be useful, and others prefer sharing advice with other people [43]. Therefore, *building relationships* is modeled as the second subdimension of functional aspects of usage adapting the MPA model to nutrition app usage. Items measuring this dimension focus on the importance of exchange with like-minded others, the support of others pursuing the same goal, and competition with other users.

**Fig. 2:**
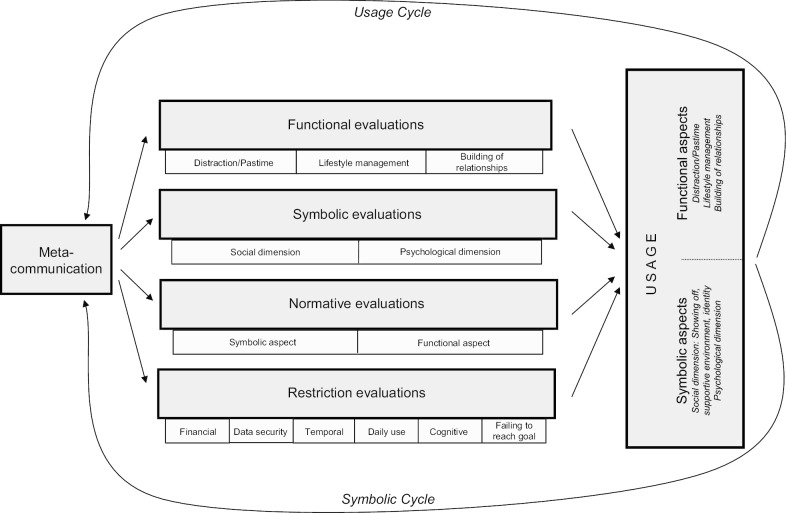
Modified version of the MPA model

Financial, temporal, and cognitive restriction evaluation were adopted from the original MPA model. Three other subdimensions were integrated according to specific challenges of app use identified by Dennison et al. [43]: (1) users may be less motivated to keep using the app if they *fail to reach their goals*, (2) it can be a burdensome task to type in information about one’s eating behavior *every day* or even several times a day, and (3) given prevailing skepticism about *data security* of health apps, this dimension was added to restriction evaluations (see Fig. 2). Items for normative evaluations were also adapted and for example, included beliefs about rules on what kind of health information should be shared on social media [43]. All items of the model were measured using 5-point Likert-type scales.

The reliability of the various subdimensions ranged from α = 0.63 to 0.80. The scale of normative evaluations, however, showed a weaker reliability, indicating that it measured more than one dimension. Based on a principal component analysis (KMO^1^ = 0.62; explained variance = 50.4%), two normative factors were identified: *subjective norms*^2^ and *public image* (see Table 1). Restrictions and the social dimension of symbolic aspects of usage were tested using single items.

**Table 1:** Statistical values and reliability of subscales (N = 761)

	Number of items	*M*	*SD*	Mean *r*_*it*_	*α*
*Meta-communication*					
Interpersonal	4	1.83	0.71	.51	.71
Mediated	5	1.46	0.45	.38	.64
Observation	4	1.42	0.51	.54	.74
*Functional evaluations*					
Distraction	2	2.97	1.15	.58	.73
Lifestyle management	3	4.38	0.68	.45	.63
Building of relationships	3	1.99	0.98	.63	.79
*Symbolic evaluations*					
Psychological dimension	4	4.30	0.70	.47	.70
Social dimension	3	2.05	0.99	.47	.66
*Normative evaluations*					
Subjective norms	3	2.06	0.80	.31	.50
Public image	3	2.81	0.95	.22	.38
*Restriction evaluations*					
Financial	1	2.39	1.42	–	–
Cognitive	1	1.49	0.90	–	–
Time	1	2.05	1.15	–	–
Daily use	1	2.43	1.32	–	–
Data security	1	2.99	1.58	–	–
Failing to reach goal	1	2.01	1.15	–	–
*Usage*					
*Functional aspects*					
Distraction	3	1.54	0.77	.64	.80
Lifestyle management	3	4.48	0.62	.45	.64
Building of relationships	3	1.52	0.81	.67	.80
*Symbolic aspects*					
Psychological dimension	4	4.06	0.67	.45	.68
Social dimension					
Showing-off	1	1.37	0.82	–	–
Supportive environment	1	2.74	1.28	–	–
Identity	1	1.89	1.22	–	–

Values measured on a 5-point Likert-type scale from 1 ‘strongly disagree’ to 5 ‘strongly agree’, resp. 1 ‘never’ to 5 ‘very often’; *mean r*_*it*_: average of corrected item-total correlation; *α*: Cronbach’s Alpha

In addition, data about the use of different features of the app MyFitnessPal and demographics were collected.

## Results

### Descriptives

Respondents had been using nutrition apps for 9.83 months on average (*SD* = 12.56). Most of them (92.4%) reported using nutrition apps several times a day with one episode mainly (74.6%) lasting between one and five minutes. The most important feature was reported as the food diary with 82.9% of respondents logging their food intake “very often”. All other features, such as step counter (37.1% “often” or “very often”) or connecting with other devices (48.9% “often” or “very often”) were used less often. As 51.5% of respondents report to “never network with friends” via the nutrition app and 49.4% reported “never using the messaging feature”, social features proved to be of minor importance.

Regarding the subdimensions of the MPA model (see Table 1), metacommunication was reported to occur only rarely among our respondents. However, they did rate high on the psychological dimension both of symbolic evaluations and symbolic use of the app. That is, although they did not talk about it, respondents were personally attached to their use of the app. Concerning both functional evaluations and uses, lifestyle management, for example, controlling weight and monitoring a healthy diet, was reported as the most important, whereas distraction and building of relationships was, on average, of nearly no relevance. The agreement with normative evaluations was low. Along with the very low occurrence of metacommunication, this low level of agreement could be a sign for an appropriation process that has only just started from a societal perspective. Most restrictions are also rated rather low; thus, our respondents did not see any major barriers to their app use. However, as we were only studying regular users of nutrition apps, this finding seems to be rather obvious. Only concerns about data privacy are rated higher, which might be a German phenomenon across all sorts of online applications [45].

### Identification of user types

In order to answer RQ1 and reveal distinct types of nutrition app appropriation, we clustered our data based on the behavioral dimensions of the MPA model as suggested by Wirth et al. [25]. Using a hierarchical cluster analysis, we identified four patterns of nutrition app appropriation among German nutrition app users: (1) Health-conscious (29.0%, *n* = 221), (2) Supported (25.0%, *n* = 190), (3) Socializer (13.5%, *n* = 103), and (4) Indifferent (29.2%, *n* = 222) as shown in Table 2. To validate our cluster solution, we conducted a multinomial logistic regression. This analysis confirmed that 96.7% of all cases were sorted into the right group based on the variables that had been used to identify the clusters (*R*^2^ = 0.92 (Cox and Snell), 0.98 (Nagelkerke), Model *χ*^2^(21) = 1830.61, *p* < 0.001). In the following section, these clusters are described based on their respective prevalent functional and symbolic aspects of usage (see Table 2). In addition, differences in terms of app functionalities used and users’ sociodemographics (see Tables 3, 4) are discussed in order to answer RQ2.

**Table 2:** Cluster description by functional and symbolic aspects of usage (N = 761)

	The Health-conscious (*n* = 221)	The Supported (*n* = 190)	The Socializer (*n* = 103)	The Indifferent (*n* = 222)	F-value
*Functional aspects*					
I’m using nutrition apps for diversion	1.41^a^	1.20^b^	2.11^c^	1.54^a^	30.1***
I’m using nutrition apps when there’s nothing else to do	1.52^a^	1.19^b^	1.94^c^	1.76^c^	20.0***
I’m using nutrition apps when I’m bored	1.64^a^	1.26^b^	1.96^c^	1.70^a^	14.9***
I’m using nutrition apps to be able to access nutritional information at any time	4.75^a^	4.28^b^	4.45^b^	3.62^c^	62.1***
I’m using nutrition apps to lead a healthy life	4.81^a^	4.49^b^	4.57^b^	4.21^c^	25.4***
I’m using nutrition apps to keep track of my nutritional status	4.94^a^	4.79^b^	4.72^b^	4.40^c^	31.3***
I’m using nutrition apps to exchange views with like-minded people	1.26^a^	1.42^b^	3.44^c^	1.18^a^	279.2***
I’m using nutrition apps to get support from others pursuing the same goal as me	1.30^a^	1.53^b^	3.83^c^	1.28^a^	338.3***
I’m using nutrition apps to compete with other users of the app	1.14^a^	1.15^a^	2.20^b^	1.07^a^	114.5***
*Symbolic aspects*					
My nutrition apps are a good fit for me	4.68^a^	4.08^b^	4.43^c^	3.34^d^	112.5***
I like using my nutrition apps	4.84^a^	4.62^b^	4.68^b^	3.99^c^	75.9***
I’m using cutting-edge nutrition apps	4.01^a^	2.92^b^	3.59^c^	2.21^d^	120.1***
I can access my nutrition apps at any time	4.85^a^	4.59^b^	4.71^ab^	3.99^c^	61.4***
Sometimes I tend to brag with my usage of cutting-edge nutrition apps	1.43^a^	1.32^a^	1.89^b^	1.10^c^	25.2***
The people close to me support my usage of nutrition apps	2.56^a^	3.69^b^	3.20^c^	1.91^d^	103.2***
Who I am is also reflected in the way I use nutrition apps	2.48^a^	1.20^b^	2.93^c^	1.40^b^	106.9***

Values measured on a 5-point Likert-type scale from 1 ‘strongly disagree’ to 5 ‘strongly agree’; ***p < 0.001, means marked by different characters differ significantly; all items were measured in German language

**Table 3:** Cluster description by sociodemographic variables (N = 761)

	The Health-conscious (*n* = 221)	The Supported (*n* = 190)	The Socializer (*n* = 103)	The Indifferent (*n* = 222)	F-value
Age	36.75^ab^	38.77^a^	33.11^c^	35.68^bc^	5.2**
Gender					
(% male)	34.4	34.4	22.5	34.7	1.8
Education					
(% high)	64.5^ab^	69.4^ac^	58.8^b^	77.2^c^	4.7**
Income per month					
(% 1500 € and higher)	59.1^a^	67.8^a^	43.0^b^	62.6^a^	4.9**
Employment					
(% employed)	74.7^a^	86.2^b^	61.6^c^	73.4^a^	7.7***

**p < 0.01, ***p < 0.001, means marked by different characters differ significantly

**Table 4:** Cluster description by use of nutrition app functionalities (N = 761)

	The Health-conscious (*n* = 221)	The Supported (*n* = 190)	The Socializer (*n* = 103)	The Indifferent (*n* = 222)	F-value
Nutrition diary	4.84^a^	4.81^a^	4.77^a^	4.54^b^	7.2***
Barcode scanner	4.04^a^	3.97^a^	3.90^ab^	3.69^b^	3.6*
Display recently used foods	4.49^a^	4.41^a^	4.43^a^	4.16^b^	6.0***
Visual display of own progress	3.91^a^	3.78^a^	3.85^a^	3.52^b^	4.7**
Edit personal profile	2.23^a^	2.18^a^	2.74^b^	1.93^c^	14.1***
Set or change nutritional goals	3.14^a^	2.99^ab^	3.40^c^	2.81^b^	6.5***
Analysis of calorie intake	4.38^a^	3.96^b^	4.02^b^	3.88^b^	9.0***
Reminder signal	2.41^ab^	2.59^ab^	2.75^a^	2.21^b^	3.0*
Step counter	3.64	3.61	3.51	3.77	0.4
Network with friends	1.82^ac^	2.03^a^	3.16^b^	1.58^c^	27.9***
Messages	1.84^a^	1.86^a^	2.46^b^	1.53^c^	10.8***
Connect with other devices	3.88	3.86	3.98	3.63	1.3
Edit privacy setting	2.77^ab^	2.70^ab^	2.99^a^	2.44^b^	3.8*
Edit own foods or meals	3.17^a^	3.14^a^	3.37^a^	2.82^b^	4.5**
Push messages	2.31^a^	2.11^a^	2.85^b^	2.00^a^	7.9***
Help pages	1.65^ab^	1.59^ab^	1.79^a^	1.45^b^	2.7*

Values measured on a 5-point Likert-type scale from 1 ‘never’ to 5 ‘very often’; *p < .05, **p < 0.01, ***p < 0.001, means marked by different characters differ significantly

#### The Supported

These users can rely on the support of their social surroundings. They reported that people close to them encouraged them to use nutrition apps. Their main motive to use nutrition apps was to keep track of their nutritional status, whereas distraction or pastime were not relevant to them. Consequently, individuals in this group used the app several times a day. Regarding sociodemographic factors, this cluster had the highest share of employed users with a high income; accordingly, the average age of users is also highest in this cluster as compared to the other clusters.

#### The Indifferent

The Indifferent use the nutrition app mainly for pastime activities; compared to the other clusters, enjoyment of using the app or access to nutrition data has only a minor role for them. Consequently, these respondents use all MyFitnessPal features less often than the other respondents. Considering sociodemographic factors, the percentage of users with high educational levels is highest in this cluster.

#### The Health-conscious

Users in this cluster are innovative and define themselves through their use of the nutrition app, which is rather functional and goal-oriented. Sociodemographic factors are on an average level within this cluster; however, a few significant differences with regards to age (older than the Socializer), income (higher income compared to the Socializer), education (lower education than the Indifferent), and employment rates (lower than among the Supported, higher than among the Socializer) were noted.

#### The Socializer

The Socializer especially values the social features of the app. People in this group like to get in touch with like-minded individuals and receive support from them but also to compete with others pursuing the same goals as they do. They also like to use the app for distraction, and they edit their personal profiles in the nutrition app more often than others. Users in this cluster report the longest average single usage episodes compared to all other clusters. Accordingly, these users do rate highest on nearly all functionalities of the app (such as maintenance of their own profiles, connecting with friends, specification of nutritional or weight goals). Furthermore, this is the youngest cluster with the highest share of women. Additionally, the number of users having a higher income and being employed is smaller than in any other cluster.

### MPA factors influencing actual usage behavior

To answer RQ3, we conducted another multinomial logistic regression. In contrast to the first regression that was computed to validate the cluster solution with the variables used to identify the clusters, we entered the behavioral factors suggested by the MPA model, namely, metacommunication, functional, normative, and restriction evaluations (see Figs. 1, 2) as predictors of the cluster affiliation in this analysis. Based on these factors, 57.9% of all cases were sorted correctly into one of the four usage clusters indicating that the behavioral predictors suggested by the MPA explain the appropriation patterns quite well (see Table 5). However, the four clusters differed with regards to their predictability. While the *Health-conscious* were best predicted by the MPA factors with 70.5% of the cases being correctly classified, only 31.2% of the *Supported* were classified correctly using these predictors.

**Table 5:** Classification of cases based on multinomial logistic regression of MPA constructs

Observed	Predicted				Percentage correct
	The Supported	The Indifferent	The Health-conscious	The Socializer	
The Supported	59	59	50	21	31.2
The Indifferent	25	146	43	4	67.0
The Health-conscious	30	26	155	9	70.5
The Socializer	13	12	14	60	60.6
Overall percentage	17.5	33.5	36.1	12.9	57.9

Based on this regression model and using the *Indifferent* as the reference group, we further assessed which MPA factors best predict the different usage patterns (see Table 6). As the results show, observation as a subdimension of metacommunication, distraction as a functional evaluation, and restriction evaluations did not significantly predict cluster affiliation. Instead, interpersonal metacommunication significantly predicted all three clusters with this influence being strongest for the *Socializer*. All other predictors were significant for only one or two of the clusters.

**Table 6:** Multinomial logistic regression of MPA predictors on cluster affiliation

	The Health-conscious versus The Indifferent		The Supported versus The Indifferent		The Socializer versus The Indifferent	
	B (SE)	Exp(B)	B (SE)	Exp(B)	B (SE)	Exp(B)
Intercept	− 12.95 (1.42)***		− 5.32 (1.05)***		− 15.63 (1.79)***	
*Metacommunication*						
Interpersonal	0.78 (0.21)***	2.18	0.73 (0.20)***	2.08	1.27 (0.27)***	3.56
Mediated	0.25 (0.30)	1.29	− 0.55 (0.30)	0.58	0.74 (0.36)*	2.09
Observation	− 0.12 (0.29)	0.89	0.24 (0.27)	1.27	0.28 (0.35)	1.32
*Functional evaluations*						
Distraction	0.07 (0.11)	1.08	− 0.14 (0.11)	0.87	0.06 (0.17)	1.07
Lifestyle management	0.85 (0.22)***	2.33	0.56 (0.19)**	1.76	0.37 (0.29)	1.45
Building of relationships	− 0.25 (0.16)	0.78	0.21 (0.15)	1.24	1.68 (0.21)***	5.38
*Symbolic evaluations*						
Psychological	1.66 (0.25)***	5.26	0.38 (0.19)	1.46	0.61 (0.32)	1.85
Social	0.27 (0.16)	1.30	− 0.18 (0.16)	0.84	0.72 (0.20)***	2.05
*Normative evaluations*						
Subjective norms	0.48 (0.19)*	1.61	0.81 (0.19)***	2.25	0.47 (0.25)	1.59
Public image	0.31 (0.13)*	1.36	0.04 (0.13)	1.04	0.09 (0.20)	1.09
*Restriction evaluations*						
Financial	0.00 (0.09)	1.00	− 0.00 (0.09)	1.00	− 0.05 (0.14)	0.95
Cognitive	− 0.18 (0.16)	0.83	0.19 (0.13)	1.21	0.09 (0.20)	1.09
Temporal	− 0.28 (0.14)	0.75	− 0.14 (0.13)	0.87	0.06 (0.20)	1.06
Daily use	− 0.08 (0.12)	0.93	− 0.15 (0.12)	0.86	− 0.18 (0.17)	0.83
Data security	− 0.08 (0.08)	0.92	− 0.08 (0.08)	0.92	− 0.12 (0.12)	0.87
Failing to reach goal	− 0.14 (0.11)	0.87	− 0.11 (0.11)	0.90	0.09 (0.16)	1.09

*R*^2^ = .54 (Cox and Snell), .58 (Nagelkerke), Model *χ*^2^(48) = 568.05, *p* < .001; *p < .05, **p < .01, ***p < .001

Being classified as a *Socializer* was more probable with interpersonal or mediated metacommunication about nutrition apps. Moreover, the importance of building relationships in addition to a positive evaluation of the social dimension increased the likelihood to be classified as a *Socializer* compared to the *Indifferent* as the reference group.

Being classified as *Supported* was predicted by three components of the MPA model: (1) interpersonal metacommunication, (2) lifestyle management as a functional evaluation, and (3) subjective norms. This subdimension of normative evaluations suggesting the notion that it is socially desirable to use nutrition apps was most important for the *Supported* compared to the other clusters.

The *Health-conscious* group was predicted by a broader range of determinants. Besides interpersonal metacommunication, lifestyle management as a functional evaluation, both dimensions of normative evaluations (subjective norms and public image), and also the psychological dimension of symbolic evaluations indicated that it was far more likely to be classified as *Health-conscious* instead of *Indifferent*. The influence of the latter indicates that the *Health-conscious* found it more important to use cutting edge nutrition apps, which suited and enabled them to always be able to access nutrition status, than the *Indifferent*.

## Discussion

Taking an appropriation perspective to examine the use of nutrition apps proved to be fertile. While our sample was rather homogenous regarding the metrics of nutrition app use (such as length of use, usage episodes per day) we identified four distinct patterns of nutrition app appropriation based on the dimensions proposed by the MPA model [25]: (1) Health-conscious, (2) Supported, (3) Socializer, and (4) Indifferent. These four types of users differ mainly with regards to (1) their willingness to adjust default settings to one’s own needs and abilities, (2) the role of social support and social norms, and (3) app use for socializing and competition. Consequently, in order to promote healthy lifestyles with mHealth, apps and communication about the app should be adjusted to the preferences and appropriation patterns of the different user types.

The Health-conscious group seemed to be well aware of the fact that keeping or achieving a healthy status is important and that the app helps to support personal lifestyle-management. Thus, use of the app is highly functional and goal-oriented. More specifically, this user type frequently uses standard features of an app that fit his/her needs while keeping track of the food intake and maintains the default settings rather than interacting with the app and changing the settings. This finding might be explained by the fact that this user group is middle-age with moderate levels of education and income; thus, these people are somewhat older and less educated than at least some of the other groups. As a consequence, digital competencies to engage in more elaborate uses of the app might also be lower in this group [46]. Therefore, app developers should strive for providing apps that are easy to use and do not demand too many digital competencies or decision-making abilities in order to achieve continuous use among this user type. Since maintenance or achievement of health plays a major role, these users don’t need to be made aware of the relevance of a healthy lifestyle but might profit from recommendations by doctors or peers for suitable and easy-to-use apps. As Rossmann et al. [34] showed in their study with diabetic patients, recommendations by doctors can be an important driver of app use. In turn, this user type will be a useful multiplier to communicate good experiences with an app to others, since interpersonal communication about the app in addition to normative and symbolic evaluations, especially the use of a cutting-edge nutrition app, are strong predictors of this appropriation pattern.

The Supported has a rather similar use pattern as the Health-conscious. Also, this user type uses the app frequently, mostly for monitoring nutrition intake, and is driven by the goal to achieve better lifestyle management and interpersonal metacommunication. The difference between these two types lies in the role of social support and subjective norms. Contrary to the Health-conscious, appropriation of the app among the Supported is strongly influenced by other people encouraging them to use the app. Since this user type involves older people with a relatively high income and employment rate and social support in addition to interpersonal communication about the app are important, it would again be advisable to encourage peers and doctors to recommend and support use of the app. Although research shows the willingness to recommend or use apps by dietetic practitioners has increased over the past years [47–49], nutrition apps are hardly used as a means for behavior change and have no central part in the nutrition care process [48]. The reluctance to recommend nutrition apps can be explained by several challenges that practitioners perceive, such as their personal lack of knowledge about the apps, low motivation, and low perceived self-efficacy but also app factors, such as low usability, app quality, and high costs [49, 50]. Thus, the recent decision to allow prescription of health apps in Germany ensuring reimbursement by statutory health insurances for apps that have been tested for safety, functionality, quality, data security, and data protection by the Federal Institute for Drugs and Medical Devices (BfArM) should be a strong driver to facilitate recommendations by physicians [51].

Although nutrition apps sometimes lack accuracy, they may provide a valuable support for nutrition management, especially when combined with guidance from dietitians [47, 52]. Using apps as one aspect of telemedicine, dietitian counseling could be integrated more easily into the therapy of chronic diseases [53]. Training programs for dietitians to learn how to facilitate integration of apps into their daily practice, especially by strengthening their self-efficacy, could enhance their willingness to do so [50]. Such training programs should also emphasize the possibility of facilitating provider–patient communication with the help of nutrition apps for two reasons: (1) app-based dietary records can facilitate automatic nutrition assessment sparing valuable time for communication about other issues, such as nutrition counseling and education [54] and (2) our results show that both for the Health-conscious and the Supported, app use might be fostered by providing the technical opportunity in addition to communicating the possibility to use the app or other communication functions of the smartphone (such as WhatsApp) for communication with doctors and peers.

The literature on mHealth effects often emphasizes the potential of health apps to facilitate interaction and exchange with like-minded peers and health experts either within the app or by connecting the app with social media platforms [55]. However, our results show that these opportunities are only relevant for a certain type and only a minority of users: Socializers (13.5%). This type frequently uses all types of app functionalities but especially features demanding interaction and change of settings (such as profile settings, specification of nutritional or weight goals, connecting with friends). Their goal is not mainly health-oriented but rather exchange and competition-oriented. Therefore, this user type is especially interested in exchanging views with like-minded people via the app, receiving support from others pursuing the same goal, and competing with others’ achievements. Due to this broader use spectrum, this type also spends more time with the app. In line with general use data for social media [56], this user type is younger than the other types and presumably has higher technical competencies [46]. Thus, this user type needs apps that provide both good monitoring and feedback features and easy ways to connect with other peers and social media. Moreover, this group should profit from gamification elements that support and enable competition, such as by offering leader boards, contests, and badges, in order to maintain the willingness to continue using the app (see systematic reviews on gamification elements in mHealth [57, 58]).

While the above described user types can be characterized by specific attributes, needs, and goals evoking specific app functionalities to be offered and ways to choose for motivation, the last and largest group of users (29.2%) can be addressed less easily. The Indifferent uses all app features less often than any of the others. When members of this type use the app, they do it mainly for distraction or pastime, while not being particularly interested in health, socializing, or symbolic aspects of usage. Hence, the risk of discontinued usage is probably highest for this group. However, since this group comprises people with a high educational level and rather high incomes and employment rates, it can be assumed that this group’s health status is also higher. Therefore, from a public health perspective the need to motivate these people to continue using the app is not as pressing. From a marketing and app developer’s perspective, it would be advisable to offer features that attract attention, raise awareness to the relevance of healthy eating, and support entertainment. These features could include push reminders, explain-it videos about nutrition, or, as mentioned previously, gamification elements.

Thus, all in all, the differences in appropriation patterns clearly show that future mHealth interventions in the area of nutrition and physical activity will have to take account of the users’ perspective in order to be able to facilitate continuous use of the app and support the attainment of health goals.

Apart from practical implications, the results also shed light on some theoretical insights. While all in all the model proved useful for detecting variability in preferences for app functionalities and functional and symbolic aspects of use, the predictability of the appropriation patterns from the model factors, namely functional, symbolic, normative, and restriction evaluations, differed. Prediction rates for the Health-conscious, the Socializer, and the Indifferent were quite high (ranging from 60.6 to 70.5%) indicating that the spectrum of postulated evaluations thoroughly captured the relevant needs and expectations that drive appropriation of a nutrition app. However, for the Supported, the prediction rate was distinctly lower with only 31.2% of the cases being correctly classified to this cluster based on the model predictors. Using the Indifferent as the reference group, the logistic regression showed that there is no one predictor that significantly and solely explains being classified as the Supported. Significant predictors of the Supported are lifestyle management, interpersonal metacommunication, and subjective norms, all of which are also (among others) significant predictors of the Health-conscious. Only for subjective norms was the beta-coefficient remarkably higher for the Supported. Since this aspect together with receiving support for using the app were crucial characteristics of the cluster, the model might need some specification on this dimension. Research in the context of the Theory of Planned Behavior (TPB) often fails to predict behavior from subjective norms [59] and may also be due to an under-specification of the concept. Considering the theory of normative social behavior [60, 61], it becomes evident that norms comprise more than just injunctive and descriptive dimensions but also depend on the collective norms imposed by the peer-group. Along this line, the MPA model could be specified to be adapted to the health app-context more successfully.

Independent from the cluster prediction, it also became apparent that some evaluations did not differentiate usage at all in this context, such as observation (as part of metacommunication), distraction (as part of the functional evaluations), and all restriction factors (financial, cognitive, and temporal). It is not surprising that observation did not make a difference in the context of health apps. Other than in the context of general mobile phone use from where the MPA model originated, the use of a specific app is not as observable as the use of a certain device. In contrast, it is interesting to note that distraction as a predictor did not play a role in differentiating appropriation patterns, considering that the Indifferent group actually uses the app for distraction more than some of the other types. This finding might be explained by the fact that health apps are not the first type of app or medium that is considered when selecting options for distraction. However, when considering actual behavior, it becomes apparent that also more instrumental apps, such as nutrition apps, can actually take the role of a distraction and pastime medium. With regards to restriction evaluations, it is important to note that our sample consisted of users only. Thus, restriction evaluation may be important as a factor that determines app adoption, i.e., use or non-use, but is less of a factor that differentiates user groups. Restrictions, such as the time required to log food intake, may hinder use of nutrition apps [47]. However, some results did indicate potential influences of users’ technical competences regarding the ability to change default settings (the Health-conscious). These factors might just not become apparent as predictors since those who don’t use all app functionalities might not be aware of the fact that they lack some abilities and thus actually are limited, not for using the app at all but for exploiting all possibilities. Against this background, future research should examine in more detail how far these predictors can and should be adjusted to the nutrition app context in order to better explain appropriation patterns.

Further research is needed also due to some limitations of our study. First, our sample was a self-selected one based on users of one specific nutrition app. Although we asked respondents to bear in mind all different kinds of nutrition apps, it is possible that participants mainly thought about MyFitnessPal and/or did not use any other nutrition app. Future studies should aim for a representative or at least more heterogeneous sample, also integrating a wider array of nutrition apps. In addition, due to the cross-sectional design of our study, we were not able to investigate the appropriation process in the long-term, thus not being able to describe the circular process as proposed or to prove any intention-behavior relationships. Future research will have to use adequate empirical designs to integrate this dynamic aspect. From a more theoretical point of view, the low reliabilities of some of the MPA subdimensions remain unsatisfactory. Future research will both have to refine the empirical measurement of these subdimensions and refine the subdimensions per se as discussed above. Especially with respect to the realm of social norms regarding the use of nutrition apps, further empirical evidence on relevant aspects is needed.

## Conclusions

This study made an important contribution to move beyond a mere description of use metrics and the binary logic of adoption of nutrition apps, thus giving a first glimpse at the multi-faceted appropriation patterns of nutrition apps. The results indicate very different user types of nutrition apps that clearly demonstrate the need for adjustments of app functionalities and usability to the demands of their users. Since not all user types are willing to adjust apps to their own needs but rather go with the default settings, a one-size-fits all app, even if allowing for adjustments, cannot be gold standard. Furthermore, app characteristics such as interaction, feedback functions, or competition that are typically discussed among the factors that increase the effectiveness of mHealth interventions, are not relevant for every user. Therefore, it is not surprising that research on the effects of mHealth fails to find consistent effects if user preferences are not taken into account. Against this background, these results pave the way for more elaborate studies on mHealth appropriation patterns and mHealth effects, as well as more fine-grained mHealth-interventions.

## Data Availability

The datasets used and/or analyzed during the current study are available from the corresponding author on reasonable request.

## Abbreviations

MPA: Mobile phone appropriation model
TPB: Theory of planned behavior
UGA: Uses and gratifications approach

## Footnotes

1 The Kaiser–Meyer–Olkin measure of sampling adequacy indicates the proportion of variance in the variables that might be caused by underlying factors. Values above .50 are acceptable [44].

2 Subjective norms refer to the perceived social pressure to engage or not engage in a behavior [26].
